# Effectiveness of a psycho-educational intervention for expecting parents to prevent postpartum parenting stress, depression and anxiety: a randomized controlled trial

**DOI:** 10.1186/s12884-020-03341-9

**Published:** 2020-10-31

**Authors:** Marjolein Missler, Annemieke van Straten, Jaap Denissen, Tara Donker, Roseriet Beijers

**Affiliations:** 1 Department of Clinical, Neuro and Developmental Psychology, Section of Clinical Psychology & Amsterdam Public Health Research Institute, Vrije Universiteit Amsterdam, Amsterdam, The Netherlands; 2 Department of Developmental Psychology, Utrecht University, Heidelberglaan 1, 3584 CS Utrecht, The Netherlands; 3 Department of Psychology, Laboratory for Biological and Personality Psychology, Albert-Ludwigs-University of Freiburg, Stefan-Meier-Straße 8, D-79104 Freiburg im Breisgau, Germany; 4 Radboud University, Behavioural Science Institute, Montessorilaan 3, 6525 HR Nijmegen, The Netherlands; 5 Radboud University Medical Center, Donders Institute for Brain, Cognition & Behavior, Nijmegen, The Netherlands

**Keywords:** Psycho-education, Universal prevention program, Randomized controlled trial, Parenting stress, Pregnancy

## Abstract

**Background:**

The first months postpartum can be challenging for parents, leading to elevated symptoms of parenting stress, depression and anxiety. In turn, distressed parents are at higher risk for providing suboptimal quality of caregiving. As psychoeducational interventions can be effective in reducing psychological distress, the goal of this randomized controlled trial was to examine the effectiveness of low-intensity universal psychoeducational program to prevent postpartum parenting stress, and to enhance parental well-being and caregiving quality.

**Method:**

Between 26 and 34 weeks of pregnancy, 138 pregnant women and 96 partners were randomized to the intervention or a waitlist control group. The intervention consisted of a booklet, a video, a home visit, and a telephone call. Information was provided on (1) sensitive responsiveness, adapting to the parental role, and attending to own needs; (2) crying patterns; (3) feeding (arrangements); and (4) sleeping (arrangements). The primary outcome was parenting stress postpartum. Secondary outcomes were additional measures of distress (depression and anxiety), parental well-being, and caregiving quality.

**Results:**

Both groups showed a rise in distress after birth. No between-group differences were observed on parenting stress, nor on the secondary outcomes. The intervention was rated as useful and of added value by the parents.

**Conclusion:**

This study offered no evidence that our universal prevention program was effective in decreasing parental distress or in increasing caregiving quality. However, parents found aspects of the intervention useful. More research is needed, including a longer period of follow-up as well as observational measures of parents’ responsiveness.

**Trial registration:**

This trial has been registered on 15 September 2016 in the Netherlands National Trial Register, ID: NTR6065, https://www.trialregister.nl/trial/5782.

The transition to parenthood is an important and challenging life event that can be accompanied by significant distress. Parents of a newborn report doubts about their own parenting skills [1] and feeling overwhelmed by the seemingly unlimited demands that come with the parenting role [2]. This results in lowered self-efficacy [3] and reduced self-esteem [4]. Moreover, these types of parenting distress seem to be associated with more general symptoms of depression, anxiety and stress in the first months after birth [3, 5]. Prevalence rates for maternal postpartum depression symptomatology range between 8 and 40% [6–9], and for postpartum anxiety symptomatology between 13 and 40% [10, 11]. Importantly, about 10% of fathers report symptoms of depression, anxiety and stress after the birth of their child as well [12, 13]. Moreover, next to compromising parents’ own health, parental postpartum psychological distress forecasts more problems in the child’s emotional, behavioural, and cognitive development [11, 14–16]. Clearly, there is a need to decrease symptoms of parenting stress and enhance parental well-being in the postpartum period.

Meta-analytic evidence indicates that brief psychoeducational interventions aimed at providing information can be effective in reducing symptoms of psychological distress [17]. Because these interventions are easy to implement and low-cost, they provide a fruitful option for universal prevention research. The primary goal of the present randomized controlled trial (RCT) was to examine the effectiveness of a brief universal prevention program to prevent postpartum symptoms of parenting stress. Parenting stress is the result of an experienced discrepancy between the demands associated with the parenting role and the available resources to fulfil these demands [18]. As parents report a need for reliable and non-judgmental information about parenting a newborn [1], we expect our psychoeducational intervention to be such a resource and to decrease parenting stress. Secondary aims of this study were to examine if the intervention was also effective in preventing general symptoms of depression, anxiety and stress and in enhancing the quality of parental caregiving.

## Parental distress and quality of care

The proposed mechanism behind the associations between parental parenting distress and child outcomes is that parental distress negatively affects the quality of parenting [19–21]. For example, distress can prevent parents from focusing their attention on, and responding in a timely and sensitive manner to their infant’s needs [21]. Parental sensitivity is important for a range of child outcomes, including the formation of a secure attachment relationship between infants and their parents, social competence, regulatory capacities, and lower stress levels (e.g. [22–28]).

Next to sensitivity, parental distress can compromise the formation of the strong affective tie from parent to infant [29], commonly referred to as the maternal or paternal bond. This bond has been defined as the tie from parent to infant that facilitates parent-infant proximity and caregiving behavior, such as warmth and sensitivity [30, 31]. Lower quality of the parent-infant bond has been related to problems in children’s socio-emotional development [29, 32]. Bonding has also been related, through parenting stress, to child executive functioning at 24 months postpartum [33]. This study found that, for both mothers and fathers, feelings of bonding negatively predicted experienced parenting stress over time. In addition, for both parents, a negative indirect effect of bonding on child executive functioning problems was found via experienced parenting stress. As parenting stress is suggested to provide the child with a more negative, less predictive, and chaotic environment, these environmental circumstances can negatively affect the child’s own stress levels and subsequent neurocognitive development, but also prevent the child from a stimulating environment necessary for executive functioning skills to develop [19].

Parental distress might also affect caregiving practices, including breastfeeding and room-sharing arrangements, which are important for infant development. For example, maternal depression and anxiety have been linked to a shorter duration of breastfeeding [34, 35]. The World Health Organization recommends exclusive breastfeeding during the first 6 months after birth [36]. Breastfeeding has important and well-established beneficial effects on the child’s physical and mental health, for example protection against of infections and diabetes, and more favorable cognitive development [37, 38]. With regard to room-sharing, the American Academy of Pediatrics (AAP) recommends that children should sleep within the same room as the parents (in a separate cot) during the first 6 months after birth [39], as parent–infant room sharing is associated with reduced rates of Sudden Infant Death Syndrome (SIDS e.g., [40, 41]). Also, the availability of the parents seems to help buffering the infant’s distress [42, 43], and facilitates the breastfeeding process [44–46]. This RCT will thus also examine the effectiveness of the universal prevention program to increase parental caregiving quality (i.e. increased parental bonding, longer breastfeeding duration, and longer room-sharing duration).

## Universal, selected and indicated prevention of parental distress

There are interventions that focus on the prevention of the development of clinical disorders (such as depressive or anxiety disorders) after birth. However, these interventions are mostly aimed at mothers with symptoms of clinical disorders (indicated prevention; e.g. [47, 48] or on mothers who belong to certain risk groups for developing a disorder (selected prevention; e.g. [49–51]. Examples of risk factors are a history of psychopathology, pregnancy complications or an infant born prematurely, adverse life events, low SES, or low social support [49–52].

Both indicated as well as selective prevention interventions have been proven to be effective in preventing depression [53–57]. However, much is unknown about the effectiveness of preventive interventions on other forms of distress beyond depression, such as anxiety and general stress [56, 58]. Furthermore, much less is known about universally applicable interventions that target all pregnant women, without pre-existing symptomatology or risk factors [56, 58]. This is important because research showed a high prevalence of parental symptomatology after birth, which extends in a more chronic level of sub-clinical symptomatology for about 20–30% of parents during the first postpartum years [52, 59]. In the absence of clear risk factors, this group of parents would not be targeted by existing (indicated and selected) preventive approaches. Since chronic sub-clinical symptomatology has also been linked to more negative child development [15], this finding suggests the importance of a universal preventive approach.

Existing interventions focus almost exclusively on the mother, instead of also including the partner. However, fathers also experience a significant degree of distress in the postpartum period [12, 13, 60] and a growing body of evidence indicates that paternal distress is also associated with problems in children’s emotional and behavioural development [61, 62]. Furthermore, including partners in interventions has been positively associated with higher breastfeeding rates at 6 weeks postpartum [63] and longer duration of the breastfeeding period [64]. Including partners seems thus to be important, not only for their own health and well-being, but also to prevent negative effects of paternal distress on infant development, as well as to enhance the quality of both parents’ caregiving.

## The current study

In summary, there is a high prevalence of both maternal [6–11] as well as paternal postpartum distress symptomatology [12, 13]. Given the associations between parental symptomatology and the quality of parenting and, subsequently, child development [16, 61, 62, 65], there is a need for preventive interventions that are applicable to all expecting parents, both mothers and fathers, independent of pre-existing risk factors or symptomatology. Moreover, while existing interventions mainly focused on depression as an outcome measure, our main focus was on parenting stress. To be able to reach a broad range of parents and to foster real-world implementation, we developed an easy accessible and low-intensity intervention that can be implemented during pregnancy. The intervention consists of an information booklet, an educational video, and a prenatal home visit during pregnancy and a phone call during the first postpartum weeks. The intervention is targeted at both mothers and fathers. We will examine gender differences in distress outcomes, as well as differences in intervention effectiveness depending on prenatal levels of distress (i.e. between mothers and fathers with relatively high versus relatively low levels of distress during pregnancy). Lastly, this intervention is implemented in a Dutch sample. While there is a lack of data on parental postpartum distress in Dutch samples specifically, no indications are found that the Dutch context is different compared to other western countries.

We expected that the intervention would reduce parenting stress, as well as symptoms of depression and anxiety. Furthermore, by psycho-educating both parents already during pregnancy, we expected parents to experience more self-efficacy and satisfaction in fulfilling their roles. By preventing symptoms of parenting stress, depression, and anxiety and stimulating parental well-being, mothers as well as fathers might perceive less problems with infant crying, feeding, and sleeping. Moreover, by psycho-educating parents about typical infant crying, feeding and sleeping patterns, we expected that parents would perceive less problems with these infant behaviors. Specifically, by psycho-educating parents about typical infant sleep patterns, including frequent night-wakings, we aimed to prepare parents for broken nights and a possible lack of sleep postpartum, and expected that parental perception of the quality and quantity of their own sleep would be enhanced. Furthermore, by preventing distress symptomatology, the quality of parents’ caregiving (including bonding, breastfeeding and room-sharing) should be enhanced.

## Materials and methods

### Trial design

We used a randomized controlled trial with two parallel groups: an intervention and a waitlist control group. We used block randomization (blocks 6–8) stratified by birth order (first child/ no first child) and participation of the partner (yes/no). An independent researcher generated random number sequences with a 1:1 ratio and allocated each participant to either the control or the intervention group. Blinding of the researchers or the participants was not possible. The control group was offered access to the intervention after the final assessment, which was scheduled 10 weeks after birth. The design of the study was described in full in [66].

### Participants and recruitment

Pregnant women and their partners were recruited between November 2016 and February 2018 through online media and midwifery practices in the Netherlands. Information about the study was provided digitally through 1) online newsletters providing information about the 26th week of pregnancy; and 2) advertisements on websites where pregnant women could find general information about being pregnant and where they could get in touch with other pregnant women. Furthermore, midwifery practices handed pregnant women a flyer with information about the study. In the online newsletters and advertisements, as well as in the flyer, a link to the website of the study was provided, through which potentially interested pregnant women could register for the study. Inclusion criteria were a) gestational age < 34th weeks; b) no severe pregnancy complications (i.e. gestational diabetes or pre-eclampsia); c) sufficient Dutch language proficiency; and d) access to the internet. Exclusion criteria were: a) current psychological treatment for psychopathology or psychological treatment in the 6 months before registration and b) the development of severe pregnancy complications during the course of the study. There were no requirements with respect to baseline distress level. All women were eligible. Pregnant women could participate with or without their partner.

### Sample size calculation

Hiscock et al. [67] reported an odds ratio of .57 with regard to the prevention of maternal symptoms of depression in the intervention group. We converted this ratio into an effect size of *d* = .48. Using a power of 80% and setting alpha at .05, this resulted in a sample size of 64 participants per condition (total *n* = 128).

### Procedure

**Fig. 1:**
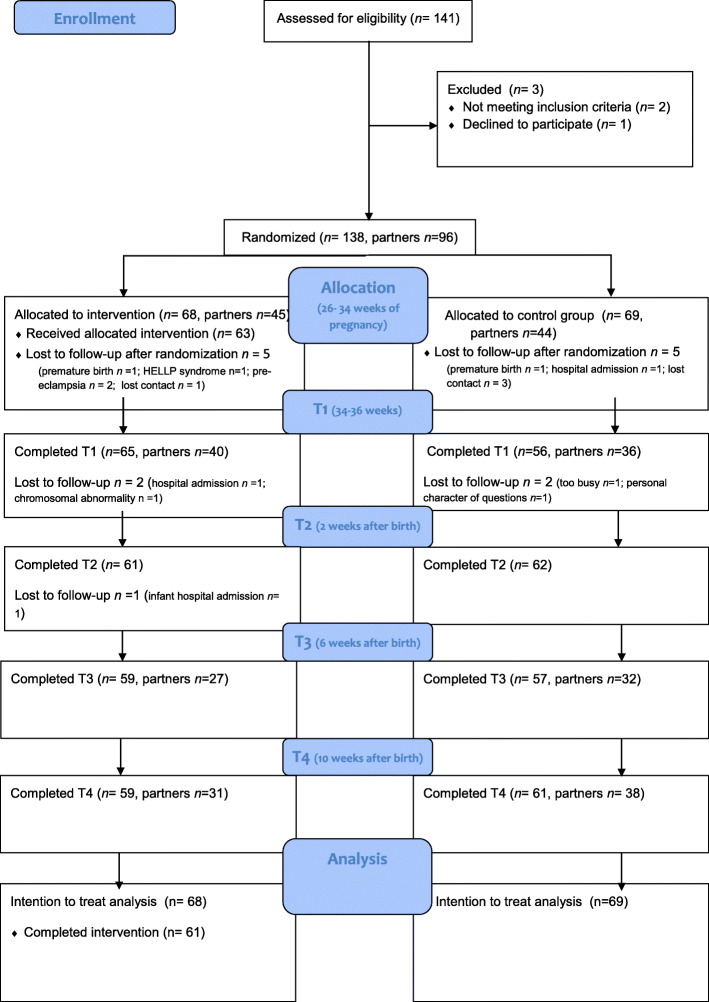
Consolidated Standards of Reporting Trials (CONSORT) flow diagram

Upon registering through the study website, interested parents received a digital information brochure of the study and an informed consent form. After providing informed consent, parents received a link to the baseline questionnaire. Upon completion, couples (or pregnant women participating without their partner) were randomly assigned to either the intervention or the control group. Online assessments took place at baseline (T0, between 26 and 34 weeks of pregnancy), 34–36 weeks of pregnancy (T1), 2 weeks after giving birth (T2), 6 weeks after birth (T3) and 10 weeks after birth (T4). At each point in time, parents received an e-mail with a link to the questionnaire (Fig. 1). All parents were also offered the possibility to fill in the questionnaires by paper-and-pencil. None of them made use of this possibility. The study was registered at the Netherlands Trial Register (NTR6065). The study was approved by the Medical Ethical Committee Brabant (NL58528.028.16/P1620).

### Intervention

The intervention consisted of (1) an information booklet; (2) an online video, (3) a prenatal home visit; and (4) a postnatal phone call. The booklet and video were received during pregnancy, after the baseline assessment (T0). Given that parenting stress and the quality of parenting can affect child development from birth onwards [68, 69], we aimed to intervene already during pregnancy. Therefore, the main part of the intervention (booklet, video, home visit) was implemented before birth. This way, there was sufficient time for parents to digest and use the information in preparing for the birth of their child.

#### Booklet and video

The booklet consisted of four chapters on (1) interpreting of, and sensitive responding to, the infant’s needs and signals of distress; as well as adapting to the parental role and attending to own needs, taking a sufficient amount of rest, and seeking support; (2) patterns of crying and different soothing techniques; (3) the infant’s hunger signals and feeding arrangements (e.g. breastfeeding, availability of professional breastfeeding courses and support services, pumping and the use of a breast pump, formula feeding); and (4) sleeping patterns and sleeping arrangements (e.g. infant sleep development and consolidation, room-sharing, bed-sharing, and solitary sleeping). We based the booklet on the work of [66], which we further developed and extended based on recent empirical research and in collaboration with academic and clinical experts [66]. The video provided illustrations of the topics described in the booklet. In the video, the experiences of upcoming parents were shown. An expert on infant development commented on the fragments, and parents were actively engaged in the video by stimulating them to think about and discuss with each other how they could implement the information within their own lives. The video was developed by psychologists with extensive knowledge on infant development and with experience in translating scientific knowledge into tools for practice (Stichting Babywerk, The Netherlands). Watching the video and responding to the questions took about 15–20 min. More details on the content of the booklet and/or the video can be found in [66].

#### Prenatal home visit and postnatal phone call

Parents were asked to read and watch the materials before the prenatal home visit. The primary aim of the home visit was to discuss the information in the materials and to respond to the parent’s questions. A secondary aim of the home visit was to explain the parents that no part of the provided information was meant to be prescriptive. Rather, we asked parents to think about how the information would fit their own and their child’s needs, and to discuss how the information could be implemented in their lives. Thus, with the home visits, we aimed to offer both education and support. Another major reason for visiting parents in their homes is to facilitate their participation in the study and in the intervention (as parents did not have to travel to a clinic or parenting class). Furthermore, by visiting parents in their homes, we expected that especially for fathers there would be less of a barrier for participating in the study [70]. All parents were visited at their homes by the first author, who has a background in clinical psychology and infant developmental psychology. About 4 weeks after the birth of their child, a phone call was scheduled. The aim of this phone call was to ask how parents and their child were doing, and to discuss possible problems in the implementation of the provided information in their lives. All phone calls were performed by the first author. Parents were given the opportunity to ask questions with regard to sensitive responsiveness and contact-seeking of the infant; and any issues in relation to the infant’s crying, feeding, and sleeping behaviors. Parents were also given the opportunity to discuss their own well-being, such as feelings of depression, anxiety or stress. Both during the home visit and the postnatal phone call, as well as in the information booklet, we explicitly described that the birth of a child is a major life change that can evoke different emotions and that adapting to the new situation takes time. We also explained that many parents can feel tired, sad or frustrated during the first months after birth. We encouraged parents to discuss their emotions and needs with their partner, and to seek additional support from their social network or support services when needed.

### Waitlist control group

Parents randomized to the waitlist control group received the psychoeducational materials after they had completed the last assessment, about 10 weeks after the birth of their child. The information was still applicable for them at that time. Also, they were given the opportunity to schedule a phone call to discuss the materials. None of the parents from the control group made use of the possibility for a phone call. All parents in the study, both in the intervention and control group, had access to care-as-usual (e.g. visits to the well-baby clinic, general practitioner) during the pre- and postpartum period.

### Measures

Online questionnaires were sent to both the mother and the father. The questionnaires assessed the primary outcome (parenting stress), as well as the other indicators of distress (symptoms of depression and anxiety) and parental well-being (satisfaction with the parenting role, parenting self-efficacy, and sleep quality and quantity). Furthermore, we measured quality of caregiving, including parent-infant bonding, duration of exclusive breastfeeding, duration of room-sharing (the infant sleeping in the parents’ room on a separate surface) and parental perception of problems with infant crying, feeding, and sleeping. We also assessed potential confounders, namely: characteristics of the delivery and birth, attachment styles of the parents, and marital satisfaction. Finally, we measured the uptake of the intervention.

#### Parental distress

##### Parenting stress

Parenting stress was assessed with 10 items of the Parenting Stress Index (PSI [71, 72];). Since the PSI has been originally developed for parents of children up to 14 years [72], we selected the 10 items that are most relevant for parents of a newborn child and added one item because it was considered central to the parenting stress construct (especially for parents of a newborn): “The responsibility I have for my children weighs on me” (see also [73]). An example of the other items is: “I feel restricted by my responsibilities as a parent.” Response options varied from 1 (totally disagree) to 6 (totally agree). A total score is derived by summing the individual item scores ranging from 11 (no stress) to 66 (very high stress). For mothers, Cronbach’s alpha in the current study was .63 (T0), .67 (T1), .73 (T3), and .75 (T4). For partners, Cronbach’s alpha was .75 (T0), .73 (T1), .75 (T3), and .84 (T4).

##### Depressive symptoms

Depressive symptoms were assessed with the Edinburgh Postnatal Depression Scale [74, 75]. This scale consists of 10 items. Participants could indicate the experienced frequency of each depression-related statement on a 4-point scale. Items are scored from 0 to 3 and the total score ranges from 0 (no depressed feelings) to 30 (severely depressed feelings). The scale shows good psychometric properties: Pop et al. [75] reported a Cronbach’s alpha of .82 and sufficient concurrent validity. Cronbach’s alpha in the current study ranged from .77 (T1) to .85 (T3) for mothers, and from .71 (T0) to .83 (T4) for partners. EPDS cut-off scores were subsequently used for descriptive purposes. To screen for (minor) depression and describe depression symptomatology in the general population, a cut-off score of 10 or more was used, as previously recommended [74, 76]. As it has been suggested that men are emotionally less expressive compared to women [77], the procedure was followed as reported by Leung, Letourneau, Giesbrecht, Ntanda, and Heart [78], and a slightly lower cut-off score was used for fathers (EPDS score of 9 or more). To be consistent and for sake of clarity, the same cut-off scores for both mothers and fathers were used in the prenatal and the postnatal period (i.e. 10 or more for mothers and 9 or more for fathers).

##### Anxiety symptoms

Symptoms of anxiety were measured with the anxiety subscale of the Hospital Anxiety and Depression Scale (HADS [79]; Dutch translation [80]). This subscale consists of seven items. For each item, participants could indicate their level of experienced anxiety on a 4-point scale. Total scores range from 0 (no anxiety) to 21 (severe anxiety). Spinhoven et al. [80] reported good psychometric properties for the Dutch version. In the current study, Cronbach’s alpha ranged from .64 (T1) to .71 (T4) for mothers, and from .73 (T0) to .76 (T1) for partners. A cut-off score of 8 or more was used to identify anxiety symptomatology within the clinical range [81].

#### Parental well-being

##### Satisfaction with the parental role

Satisfaction with the parental role was measured with three items of the Dutch translation of the Parenting Stress Index (PSI [72, 82]). As has been done before, four items were added to the scale (see [66, 73]). The addition of these items was necessary because to our knowledge, no measure of parents’ satisfaction with their new role currently exists. An example item is: “I enjoy spending time with my child.” Cronbach’s alpha in this study ranged from .64 (t0) to .76 (t3) for mothers, and from .77 (t4) to .89 (t1) for partners.

##### Self-efficacy

Self-efficacy was assessed with 1 item, through which parents could indicate their (expected) efficacy as a parent on a 5-point scale, ranging from 1 (not very good) to 5 (a very good parent [66, 82].

##### Perception of squality and quantity

Perception of sleep quality and quantity were measured with two items of the Pittsburgh Sleep Quality Index (PSQI [83]; see also [84]). The items were phrased as follows: ‘Over the last 2 weeks, how would you rate your own sleep quality/sleep quantity?’. Parents could indicate their experienced sleep quality on a 4-point scale, varying from ‘Not nearly good enough’ to ‘More than good enough’. Because of high correlations between quality and quantity at T3 (r = .61, *p* < .05), and at T4 (r = .67, *p* < .05), and our desire to reduce the number of analyses, sleep quality and quantity scores were standardized and subsequently averaged across the two time points.

#### Quality of caregiving

##### Bonding

Bonding between parent and child was measured with the Maternal Postnatal Attachment Scale (MPAS [85, 86]). This scale consists of 19 statements, for example: “When I am with the baby, I feel tense and fearful.” Parents could indicate how much they agreed with each statement on a 2-point; 4-point; or 5-point scale. By summing up all items, a total score between 19 and 95 can be reached. Lower scores are an indication of bonding problems between parent and child. When administered between 2 and 3 months after birth, Van Bussel et al. [86] reported a Cronbach’s alpha of .75. In the current study, Cronbach’s alpha was .81 for mothers (T4) and .86 (T4) for partners.

##### Breastfeeding

The duration of exclusive breastfeeding was assessed by asking mothers to indicate the number of weeks their child received exclusive breastfeeding, and thus no formula.

##### Room-sharing

Mothers were asked to indicate the number of weeks of room-sharing, defined as the infant sleeping in the room of the parents at night (in a separate cot or in the bed of the parents).

##### Perception of problems with infant sleeping, crying, feeding

Additionally, both mothers and fathers were asked in the online questionnaire (t3 and t4) whether they had experienced a problem with infant sleeping, crying, or feeding (at day and/or at night). If they responded affirmatively, they were asked to indicate the severity of this problem on a 7-point Likert scale. Response options varied from 1 (hardly any problem) to 7 (a severe problem) (see also [67]).

#### Confounding variables

We included the following confounding variables and tested for between-group differences on these variables. In case of non-significant results, we did not include the variable in our main analyses.

##### Birth characteristics

Characteristics of the delivery and birth were assessed by parents’ self-report and included birth weight, Apgar score at 5 min, and spontaneous delivery versus caesarean section.

##### Attachment style of the parents

To control for possible insecure attachment styles of the parents (which can impact on their caregiving quality [87, 88];, parental attachment style was measured with the short form of the Experiences in Close Relationships Questionnaire (ECR- short form [89]; Dutch translation [90]). The 12 items of this instrument are derived from the avoidance and anxious attachment subscales of the ECR-R (six items of each subscale [91]). Response options vary from 1 (strongly disagree) to 7 (strongly agree). The avoidance subscale measures the need to stay independent from others and to avoid intimacy [89], see also [92]. The anxiety subscale measures the degree to which the subject worries about rejection and abandonment [89, 92]. Following recoding of items 15, 25, 27, 29, and 31; for each subscale (anxiety and avoidance) an average score of between 1 and 7 can be computed. Higher scores reflect more attachment anxiety and avoidance. The ECR-short form showed good psychometric properties in different samples: Lafontaine et al. [89] reported Cronbach’s alpha’s of .78 to .87 for the anxiety subscale and .74 to .83 for the avoidance subscale. In the current study, for mothers, Cronbach’s alpha was .76 for the anxiety subscale and .85 for the avoidance subscale. For partners, these values were .76 (anxiety) and .85 (avoidance).

##### Marital satisfaction

Participants’ satisfaction with their relationship was measured with the global satisfaction items of The Investment Model Scale (IMS [93]; Dutch translation: [94]). This scale consists of five items, with answering options varying from 1 (totally disagree) to 9 (totally agree). An example item is: “My relationships fulfills my needs for intimacy.” The total score ranges from 5 (not satisfied) to 45 (very satisfied). Montgomery et al. [94], reported a Cronbach’s alpha of .93.

##### Intervention uptake and satisfaction

The uptake of the intervention was measured by asking parents whether they had read and watched the materials before the birth of their child. We also asked them whether they looked into the materials again after the birth of their child. Furthermore, we asked them to rate the frequency of using the information in their daily lives, with the item: “How many times did you use the information from the booklet, video, or the home visit during the daily care for your baby?” Response options were: “Daily”; “Several times a week”; “About once a week”; “About once every 2 weeks”; “About once a month”; and “Never.” We also asked them to rate the usefulness of the booklet, video, and the home visit on a 5-point scale ranging from 1 “Not very useful” to 5 “Very useful.”

### Statistical analyses

#### Preliminary analyses

We investigated the effect of the intervention on all different outcomes. First, we inspected distress scores over time for men and women separately (Tables 2 and 3). We analyzed whether there were between-group differences at the follow-up (T3) and final assessments (T4) for all distress (parenting stress, depression, anxiety), well-being (self-efficacy, satisfaction with the parenting role) and quality of caregiving outcomes (bonding, duration of breastfeeding, duration of room-sharing, perception of infant problems).

Because of high intercorrelations, scores at T0 and T1 were averaged for parenting stress (*r* = .75; *p* < .01 and *r* = .77; *p* < .01 for partners), depression (*r* = .66 for mothers and .62 for partners; *p* < .01), anxiety (*r* = .58 for mothers and .70 for partners; *p* < .01), satisfaction with the parenting role (*r* = .45 for mothers and .38 for partners; *p* < .01), and self-efficacy (*r* = .61 for mothers and .58 for partners; *p* < .01).

#### Main analyses

To deal with the nested nature of the data (mothers and fathers in couples), we used multilevel linear modelling (MLM). MLM is robust for missing data and is unaffected by unequal number of data points per unit, in this case the mother-father dyad [95]. Therefore, there was no need to control for the fact that more mothers than fathers participated. We could run the analyses on the full data set, including data from participants with incomplete data. As we hypothesized that parenting stress and parental mental problems would be highest around 6 weeks of infant age (i.e. the infant crying peak [96]) analyses were focused on the outcomes at T3. To test the robustness of the results, we repeated all analyses for T4.

MLM is based on a set of regression equations. First, the intercepts-only model (a model without predictors) was ran to check whether a multilevel model was required, by means of the intraclass correlation. The intraclass correlations ranged between .20 and .40, thus MLM analyses were appropriate for all variables. Second, following [95], a build-up strategy was used. Variables were added one by one to the intercept-only model. After each addition, the − 2 log likelihood ratio scale after generalized least square estimation was examined. The − 2 log likelihood tracks model fit. If model fitness increases, the added variable is kept. If model fitness decreases, the added variable is cut from the model.

The variables were tested in a certain order. First, gender (mother or father) was included as a fixed factor, and then as a random factor. Thereafter, intervention condition was added. Finally, interaction terms between intervention X parental gender, and between intervention X prenatal state (including the main effect of prenatal state) were added. These interaction terms tested whether intervention effects differed between mothers and fathers, and between parents varying on prenatal levels of the outcome of interest (e.g. whether the intervention was effective in decreasing postpartum parenting stress symptoms depending on their levels of prenatal parenting stress). Also, the interaction between intervention X parity was of interest to investigate whether the intervention was effective for first-time parents versus experienced parents (i.e. parents with children). As the majority of our sample (91.2%) consisted of first-time parents, multilevel analyses were repeated in the sample with first-time parents only. The final models are presented in the results. All analyses were done using SPSS 25.0.0.

## Results

### Descriptives

We included 138 women (68 in the intervention group and 69 in the control group) and 96 partners (48 in the intervention and 48 in the control group). Figure 1 shows the CONSORT flow diagram. The response rate of the mothers was high: 88.3% (T1); 89.7% (T2), 84.7% (T3) and 87.6% (T4). Non-response was mainly caused by medical complications during pregnancy or birth (Fig. 1). For partners, the response rate was also high: 88.4% (T1), 100%^1^ (T2); 74.7% (T3) and 85.2% (T4).

The mothers were on average between 32 and 33 years of age (Table 1). Partners were slightly older, with a mean age of about 35 years. The majority of the mothers (94.8%) were either married or cohabitating. Most participants received higher vocational education and about one third (34.7%) of mothers worked fulltime at baseline (during pregnancy). More than half of the participants reported a net family income of more than 4000 euros per month. The majority (91.2%) of participating mothers was pregnant of their first child. No significant differences between the intervention and control group emerged on the demographic variables.

**Table 1:** Demographics and confounder variables

		Mothers			Partners	
	Intervention group (*n* = 68)	Control group (*n* = 69)	*p*-value	Intervention group (*n* = 45)	Control Group (*n* = 44)	*p*-value
Age (mean, SD)	32.69 (3.37)	32.23 (3.54)	0.44	35.03 (4.08)	34.73 (5.67)	0.79
% married or cohabitating	94.1	95.6	0.51	95.5	97.7	0.27
% ≥ higher vocational education	91.1	91.2	0.51	86.7	77.2	0.31
Working hours (weekly)			0.13			0.13
employed ≥37 h (%)	28.3	39.8		57.8	72.8	
employed 21–36 h (%)	65.5	54.4		35.5	22.7	
employed 0–20 h (%)	1.5	4.4		4.4	1.5	
no paid employment	4.5	1.5		2.2	2.3	
Family income^a^ (% ≥ 4000 euro)	52.2	60.2	0.40	57.8	56.8	0.78
Birth order^a^ (% 1e kind)	91.2	91.3	0.96	88.9	84.1	0.45
# weeks pregnant at inclusion	28.24 (2.60)	27.81 (4.18)	0.45	28.20 (2.49)	27.56 (4.61)	0.39
Attachment style: avoidance	11.52 (5.82)	10.52 (3.93)	0.25	13.52 (6.54)	12.78 (5.31)	0.57
Attachment style: anxiety	16.64 (5.35)	17.24 (5.79)	0.54	17.49 (6.68)	17.44 (5.73)	0.97
Marital satisfaction	30.97 (4.39)	31.63 (3.72)	0.35	30.96 (3.87)	30.93 (3.49)	0.97
% cesarean section	9.9	11.3	0.69			
Birth weight	3484.84 (472.94)	3484.57 (424.02)	0.99			
Apgar score 5 min (% ≥7)	98.4	98.3	0.23			
Apgar score 5 min	9.43 (1.3)	9.66 (.81)				

^a^differences between mothers and partners because not all partners participated in the study

### Treatment adherence and drop-out

Of the 68 mothers who were allocated to the intervention group, 63 received the intervention (booklet, video, and home visit). The other mothers (*n* = 5) were not able to receive the intervention because of pregnancy complications developed after inclusion (i.e. HELLP syndrome or premature birth). Two mothers dropped out of the study after the home visit because of hospital admission and chromosomal abnormality (Fig. 1). In the control group, 5 women dropped out right after randomization: two because of pregnancy complications and three because they could not be contacted anymore. Two more women dropped out after T1 because they indicated they felt they were too busy to continue participation (*n* = 1) or did not feel comfortable sharing personal information *(n = 1)*.

### Parental distress

Table 2 shows the distress scores over time for mothers. As can be seen from the mean scores, both the intervention and control group showed a rise in distress (parenting stress, depression, and anxiety) and a decrease in well-being (satisfaction with the parenting role and self-efficacy) from T0 to T3, after which distress levels returned to baseline at T4. There were no significant differences on the distress variables between the intervention and control group at 6 (T3) or 10 weeks after birth (T4).

**Table 2:** Stress, depression, anxiety, satisfaction, self-efficacy and bonding mean scores for mothers in intervention and control group over time (mean; standard deviation), and *p*-values for group differences at T3 and T4

	T0 (26–34 weeks pregnant)	T1 (34–36 weeks pregnant)	T3 (6 weeks after birth)	T4 (10 weeks after birth)	*p*-value* T3	*p*- value* T4
Primary outcome						
Stress						
Intervention	32.97 (6.75)	32.91 (7.15)	39.03 (3.08)	32.42 (8.43)	.72	.22
Control	33.01 (6.25)	32.73 (6.92)	38.59 (3.48)	30.59 (7.57)		
Secondary outcomes						
Depression						
Intervention	4.48 (3.08)	4.44 (3.60)	5.53 (4.02)	4.82 (4.20)	.86	.59
Control	4.86 (3.83)	3.87 (2.60)	5.67 (4.40)	4.44 (3.34)		
Anxiety						
Intervention	5.10 (2.10)	5.59 (2.29)	5.69 (2.53)	5.64 (2.77)	.47	.78
Control	5.35 (2.14)	5.05 (1.93)	6.05 (2.83)	5.50 (2.56)		
Satisfaction						
Intervention	38.39 (3.52)	38.82 (3.67)	32.84 (8.61)	39.40 (2.41)	.46	.71
Control	38.32 (3.41)	38.93 (3.25)	32.31 (7.33)	39.21 (2.91)		
Parental self-efficacy						
Intervention	3.94 (.52)	4.05 (.45)	3.88 (.65)	3.98 (.53)	.68	.99
Control	3.87 (.54)	3.98 (.53) 3.83 (.70)		3.98 (.53)		
Sleep quality						
Intervention	n/a	n/a	2.41 (.70)	2.58 (.74)	.15	.68
Control	n/a	n/a	2.59 (.56)	2.73 (.61)		
Sleep quantity						
Intervention	n/a	n/a	2.41 (.70)	2.58 (.74)	.70	.23
Control	n/a	n/a	2.59 (.56)	2.73 (.61)		
Parent-infant bonding						
Intervention	n/a	n/a	n/a	66.15 (6.02)	n/a	.83
Control	n/a	n/a	n/a	66.55 (5.18)		

At baseline, 5 mothers in the intervention group (3.3%) scored above the cut-off for depression (10 or more on the EPDS) and 8 mothers in the control group (12.1%). With regard to anxiety, in the intervention group, 11 mothers (16, 4%) scored 8 or more on the HADS at baseline and 14 mothers in the control group (20,6%). Ten weeks after birth (t4) 5 mothers in the intervention group (9.1%) scored above the threshold for depression, and 5 mothers in the control group (8.2%). A total of 7 mothers in the intervention group (12.7%) scored above the cut-off for anxiety (8 or more on the HADS) at t4, and 13 mothers in the control group (21.7%).

Table 3 shows the different scores over time for fathers. Also here, both groups showed a rise in distress levels and a decrease in well-being from t0 to t3, after which distress levels returned to baseline. There were no significant differences in distress between the intervention and control groups at 6 (t3) or 10 weeks after birth (t4).

**Table 3:** Stress, depression, anxiety, satisfaction, self-efficacy, and bonding mean scores for partners in intervention and control group over time (mean; standard deviation), and p-values for group differences at T3 and T4

	T0 (26–34 weeks pregnant)	T1 (34–36 weeks pregnant)	T3 (6 weeks after birth)	T4 (10 weeks after birth)	*p*-value T3	*p*-value T4
Primary outcome						
Stress						
Intervention	25.38 (6.74)	26.55 (6.58)	38.82 (3.27)	28.66 (9.66)	.61	.59
Control	24.49 (7.08)	25.67 (6.67)	38.54 (4.35)	27.36 (9.56)		
Secondary outcomes						
Depression						
Intervention	3.62 (3.20)	2.70 (3.07)	2.93 (2.62)	3.21 (3.67)	.38	.54
Control	3.93 (2.56)	3.00 (2.29)	3.66 (3.61)	3.75 (3.40)		
Anxiety						
Intervention	6.24 (3.26)	5.48 (2.86)	5.25 (2.07)	5.55 (3.11)	.73	.96
Control	5.79 (2.28)	5.86 (2.60)	5.49 (3.02)	5.58 (2.35)		
Satisfaction						
Intervention	36.76 (5.42)	36.68 (6.11)	28.61 (7.26)	38.86 (3.29)	.78	.58
Control	37.67 (3.22)	37.72 (3.48)	27.60 (7.99)	38.36 (3.48)		
Self-efficacy						
Intervention	4.11 (.65)	4.22 (.62)	4.11 (.57)	4.00 (.71)	.27	.60
Control	4.02 (.56)	4.22 (.59)	3.94 (.59)	3.92 (.55)		
Sleep quality						
Intervention	n/a	n/a	2.75 (.59)	2.93 (.66)	.22	.18
Control	n/a	n/a	2.54 (.70)	2.69 (.71)		
Sleep quantity						
Intervention	n/a	n/a	2.64 (.68)	2.86 (.85)	.39	.42
Control	n/a	n/a	2.49 (.74)	2.69 (.75)		
Parent-infant bonding						
Intervention	n/a	n/a	n/a	64.46 (6.42)	n/a	.73
Control	n/a	n/a	n/a	63.78 (6.73)		

At baseline, 4 fathers in the intervention group (8.9%) scored above the cut-off score for depression (9 or more on the EPDS) and 2 fathers in the control group (4.65%). With regard to anxiety, 13 fathers in the intervention group (28.9%) scored 8 or more on the HADS at baseline and 11 fathers in the control group (25,6%). Ten weeks after birth (t4), 4 fathers in the intervention group (13.8%) scored above the threshold for depression, and 5 fathers in the control group (13.9%). A total of 7 fathers in the intervention group (24.1%) scored above the cut-off for anxiety (8 or more on the HADS) compared to 7 fathers in the control group (19.4%).

### Multilevel analyses

The final multilevel models are presented in Table 4. For both maternal and paternal parenting stress at t3, the multilevel analyses indicated no effect of the intervention. Similarly, the interaction terms intervention X parental gender, and intervention X prenatal state did not significantly improve model fit. The intervention was thus not effective in decreasing parenting stress. Moreover, multilevel analyses indicated no effect of the intervention on parental depressive symptoms, anxiety symptoms nor on any other parental secondary outcome (satisfaction with the parenting role, self-efficacy, and perception of sleep quality and quantity). The interaction terms were also not significant, indicating that the intervention was not effective for both mothers and fathers, or for parents varying in their prenatal levels on the outcome of interest.

**Table 4:** Estimates for the best fitting multilevel models

	Estimate	SE	*p*
**Primary outcome: parenting stress (T3)**			
Intercept	14.22	3.18	< 0.001
Gender (1 = mother, 2 = father)	−.41	.96	.67
Prenatal parenting stress^a^	.57	.07	< 0.001
Deviance: 1165.48			
**Secondary outcomes**			
**Depressive symptoms (T3)**			
Intercept	4.58	.83	< 0.001
Partner (1 = mother, 2 = father)	−1.69	.46	< 0.001
Prenatal depressive symptoms^a^	.63	.09	< 0.001
Deviance: 926.42			
**Anxiety symptoms (T3)**			
Intercept	3.26	.65	< 0.001
Partner (1 = mother, 2 = father)	−.81	.36	.03
Prenatal anxiety symptoms^a^	.66	.08	< 0.001
Deviance: 799.03			
**Satisfaction with the parenting role (T3)**			
Intercept	19.96	3.08	< 0.001
Partner (1 = mother, 2 = father)	.30	.48	.53
Prenatal levels of satisfaction^a^	.48	.08	< 0.001
Deviance: 911.98			
**Self-efficacy (T3)**			
Intercept	1.38	.36	< 0.001
Partner (1 = mother, 2 = father)	.04	.09	.68
Prenatal levels of self-efficacy^a^	.61	.09	< 0.001
Deviance: 305.34			
**Perception of sleep (T3)**^b^			
Intercept	−.40	.18	.03
Partner (1 = mother, 2 = father)	.29	.13	.02
Deviance: 458.38			
**Bonding (T4)**^b^			
Intercept	67.92	1.94	< 0.001
Partner (1 = mother, 2 = father)	−.12	.96	.90
Deviance: 1141.44			

^a^prenatal state levels are averaged across T0 and T1 because of high intercorrelations (ranging between .38 and .77)

^b^no prenatal levels available

Additionally, the multilevel analyses indicated that mothers had higher levels of postpartum depressive and anxiety symptoms, and worse perceptions of their sleep quality and quantity, compared to fathers. Furthermore, for all outcomes of interest, prenatal levels were predictive of postpartum levels, indicating that symptoms were to some extent stable over the course of childbirth. Repeating the multilevel analyses with the outcomes measured at infant age 10 weeks (T4), and on the sample including only first-time parents yielded similar results.

#### Quality of caregiving

##### Bonding

The multilevel model for parent-infant bonding is presented in Table 5. For both maternal and paternal bonding with the infant at T3, the multilevel analyses indicated no effect of the intervention. Similarly, the interaction terms intervention X parental gender, and intervention X prenatal bonding did not significantly improve model fit. The intervention was thus not effective in improving parent-infant bonding.

##### Breastfeeding and room-sharing

Independent samples t-tests indicated that there were no differences between the intervention (M = 7.66, SD = 4.19) and the control group (M = 7.26, SD = 4.52) with regard to the mean duration (in weeks) of exclusive breastfeeding, *p = .78* (Table 5). Also, no differences emerged in the number of weeks participants in the intervention group (M = 8.64, SD = 3.52) and in the control group (M = 7.26, SD = 4.52*)* slept in the same room as their child at night (while the child was in his or her own cot), *p = .77*.

**Table 5:** Mean number of weeks of exclusive breastfeeding and the mean number of weeks the child slept in the same room as the parents (in own cot) at 10 weeks after birth (t4)^a^

	Intervention	Control	*p*-value
# weeks exclusive breastfeeding	7.66 (4.19)	7.26 (4.52)	.78
# weeks room-sharing	8.64 (3.52)	9.12 (3.28)	.77

^a^For room-sharing, only parents that slept with their child in the same room, while the child was in his or her own cot were taken into account (control, *n* = 51/ intervention, *n* = 50)

##### Perceived problems with infant crying, feeding, and sleeping

There was one significant difference with regard to perceived problems with infant crying, feeding, or sleeping: 16.2% of mothers in the intervention group reported problems with infant feeding during daytime at 6 weeks after birth, versus 6.9% in the control group; *p = .05*). No other differences between the intervention and the control groups emerged in the percentage of mothers reporting problems with the infant’s sleeping, crying, or feeding (assessed separately for day- vs. night-time) at 6 weeks after birth (T3) nor at 10 weeks after birth (T4; Table 6).

**Table 6:** Percentage of mothers reporting problems with the infant’s sleep, crying or feeding

	Intervention T3 (6 weeks after birth) % reporting problem	Control T3 (6 weeks after birth) % reporting problem	*p*-value T3	Intervention T4 (10 weeks after birth) % reporting problem	Control T4 (10 weeks after birth) % reporting problem	*p*-value T4
Infant sleep (night)	29.3	32.8	.69	14.5	15.0	.95
Infant sleep (day)	50.0	36.8	.13	34.5	35.0	.96
Infant crying (night)	15.5	11.6	.79	5.5	3.3	.58
Infant crying (day)	31.0	34.5	.69	27.3	21.7	.48
Infant feeding (night)	13.8	5.2	.11	3.6	6.7	.47
Infant feeding (day)	16.2	6.9	.05*	16.4	10.0	.31

##### Intervention uptake and satisfaction

Almost all mothers (98.5%) read the information booklet and watched the video before the birth of their child. For partners, these percentages were 85.7% (information booklet) and 92.9% (video). More than half of the mothers (55.6%) and about one third of the partners (32.1%) reported to have used the information after the birth of their child daily or several times a week. Moreover, participants reported the intervention to be useful. Both mothers and fathers found the information booklet the most useful part of the intervention (Table 7). The home visit and the video were rated as partly useful by both parents.

**Table 7:** Means and standard deviations of intervention usefulness as reported by mothers and fathers (as indicated on a 1–5 Likert scale)

	Mothers	Partners
Information booklet	4.15 (.87)	3.64 (.91)
Video	3.08 (1.00)	2.71 (1.05)
Home visit	3.11 (.93)	2.96 (.92)

## Discussion

The aim of this randomized controlled trial was to examine the effectiveness of a brief psychoeducational intervention to prevent postpartum parenting stress, to decrease symptoms of depression and anxiety, and to enhance parental well-being and the quality of caregiving behavior. The intervention was aimed at a universal population of parents (regardless of risk factors or (previous) symptoms), and the intervention was targeted at both parents. For both groups, there was a rise in distress scores between baseline and 6 weeks postpartum. No differences emerged in levels of parenting stress between the intervention and control group over time. This means that parents that received and digested the information and were visited at home during pregnancy did not report lower levels of parenting stress compared to parents that did not receive this support. Also, there was no effect of the intervention on symptoms of depression and anxiety, nor on the indices of parental well-being (satisfaction with the parenting role, self-efficacy, and sleep quality and quantity). With regard to quality of caregiving, no differences emerged in the quality of the parent-infant bond, nor in the duration infants received breastfeeding or slept in the parent’s room at night-time. Parents from the intervention group did not report *less* problems with their infant’s crying, feeding, or sleeping. In contrast, mothers from the intervention group reported *more* instead of less problems with infant feeding at 6 weeks postpartum than mothers from the control group.

While psycho-education has been indicated valuable in reducing symptoms of psychological distress [17], also when implemented during pregnancy [97], visiting upcoming parents at home and providing them with information about adapting to the parental role, and infant crying, feeding, and sleeping (arrangements) seemed not to be effective in preventing postpartum distress and enhancing caregiving quality. However, the intervention seemed to fill a gap in the information and tools that are currently available for parents-to-be. Parents rated the intervention as useful and of added value. The question arises how an intervention that is rated as useful, particularly by the mothers, did not result in any changes in our dependent variables. Several factors could have played a role here. First, it could be that the effect of the intervention becomes visible later on during the first year (i.e. after the initial 10 weeks). Second, it is possible that the intervention is effective on other measures that we did not take into account in this study (e.g. observed sensitive responsiveness; infant well-being). Third, the intervention might be more effective for specific groups of parents (i.e. our sample was relatively well-educated, reporting high SES). Also, we lack information on participants psychosocial history. Finally, it is possible that the intervention has no added value. We will review each of these possibilities below.

First, we followed parents until 10 weeks after birth. We expected parental distress levels to be highest within these first 10 weeks, because infant crying rises until 6 weeks and gradually decreases thereafter [96]. Indeed, in both groups, distress scores over time showed a peak at 6 weeks, and returned to baseline at 10 weeks after birth. It could be that the tools we provided parents with have a buffering effect on other peaks of parental distress during the first year. This reasoning is in line with Hiscock et al. [67] who found an effect of their psychoeducational intervention implemented shortly after birth on primary caregiver’s (in 99.6% of cases the mother) depressive symptoms at 6, but not at 4 months postpartum. Moreover, effects of the intervention on breastfeeding and room-sharing might also become visible later in the first year of life. While not leading to group differences during the first weeks postpartum, when many parents are breastfeeding and room-sharing, differences in total breastfeeding and room-sharing duration might become visible after this period. Future studies into universal prevention during pregnancy should consider to extend the study period to examine whether effects of interventions emerge later in the first year of the infant’s life.

Second, it is possible that the intervention was not effective in decreasing parental distress or increasing caregiving quality measures but impacted other measures such as observed parental caregiving quality, parental stress physiology, or infant behavior. Given that mothers from the intervention group reported more problems with infant feeding, it is possible that the intervention increased maternal awareness for potential problems, making them more prone to report these. However, no differences emerged with regard to crying and sleeping. Measuring parent-infant interaction at various moments during the first year would shed more light on whether the intervention supports parents in developing these skills. Also, our study relied heavily on self-report measures, which are known for their problems with social desirability [98]. Parental physiological stress measures, such as cortisol measurements in hair, could give insight in parental stress physiology. Also, direct measures of infant behavior should be added, such as a registration of infant crying patterns, to monitor potential effects of the intervention on infant behavior [99].

Third, our sample mainly consisted of relatively highly educated first-time parents. These parents are more likely to seek and find information about pregnancy and childrearing themselves. It is possible that less educated parents have less resources to find the necessary information and would benefit more when this information would be consequently provided by midwives and nurses, for example through a clear and brief information booklet as provided in the current study. However, as Henshaw et al. [1] showed among a sample of parents varying in income, education, and ethnicity; having access to much information does not necessarily reduce stress. There is especially a need for reliable and evidence-based information [2]. Therefore, we aimed to provide parents with up to date and scientifically validated information about the first months postpartum, and intervention effects were expected also among highly educated parents.

Related to the previous point is that we have no information about the participants psychosocial history, while it is possible that the intervention is (only) effective for parents with a history of psychosocial problems. In future research, psychosocial history should be added as a moderator of intervention effectiveness. Other risk factors that have been shown to moderate postpartum distress symptomatology could also determine intervention effectiveness, for example delivery complications [11], and relational problems [100]. In sum, it could be that a more mixed sample of parents, including less highly educated parents, as well as a sample of parents varying in risk factors, would generate different results. On a related note, while there were no between-group differences, gender differences emerged with regard to depression, anxiety, and sleep at 6 weeks postpartum. Mothers reported higher levels of symptomatology than fathers. This is in line with earlier research [59, 101]. Previous research into depression suggested that the rise in symptomatology fathers experience after birth takes place later in the first year and develops more slowly than is the case for mothers [59]. Future studies following parents longitudinally could shed more light on whether mothers and fathers need different types of support, and/or at different times during the perinatal period.

Finally, while absence of evidence is not evidence of absence, we should also acknowledge the possibility that a brief psychoeducational intervention aimed at providing information is not sufficient to influence parents’ levels of distress and their caregiving quality. It might be that interventions incorporating more sessions, as well as state-of-the-art therapeutic elements, are needed to prevent parents from developing symptomatology. For example, Haga, Drozd, Lisoy, Wentzel-Larsen, and Slining [102] reported an effect on depressive symptoms of an online intervention among a universal population of pregnant women at 6 weeks postpartum. This extensive intervention consisted of 44 online 10-min sessions and incorporated a mix of elements of cognitive-behavioural therapy, mindfulness, psychoeducation, meta-cognitive therapy, acceptance and commitment therapy, and positive psychology. Thus, this intervention differed from ours with regard to the content (psychoeducation combined with elements of a range of therapeutic techniques); the relatively high frequency of (short) sessions, and the fact that all sessions could be followed online. These factors could explain why the intervention was successful in reducing depressive symptoms, at least during the first weeks after birth.

### Strengths and limitations

This randomized controlled trial has several strengths. First, while most studies focusing on the prevention of psychopathology after birth included mothers who already displayed symptomatology (indicated prevention [47, 48]; or on mothers belonging to risk groups for developing postpartum psychopathology (selected prevention [49–51], in this study a universally applicable intervention was tested. In contrast to indicated or selected prevention, much less is known about the effectiveness of universal prevention during pregnancy [56, 58]. Second, while the majority of previous intervention focused on mothers only, we were able to include mothers as well as a substantial part of fathers. Third, while most prevention studies focused on depression as an outcome measure [56, 58], we included a variety of distress outcomes, including stress related to the parenting role and symptoms of anxiety.

Of course, the study has limitations as well. First, while we aimed to include a sample of expecting parents varying in demographic characteristics and backgrounds, our sample was relatively well-educated, and reported a relatively high income. This decreases the generalizability of this study. Notably, to be able to digest the information booklet and the video, parents needed to be able to read Dutch and have access to the internet. Since 98% of Dutch households have access to the internet at home [103], we assume that this criterion has not lead to the exclusion of lower educated parents. Instead, it seems more likely that higher SES parents actively search for and make use of this type of interventions. Indeed, a study investigating predictors of eHealth usage found that people with a lower socio-economic status were less likely to engage in a number of eHealth activities compared to their counterparts with higher socio-economic status [104]. It is important that future studies assess the reasons for these differences in the implementation of eHealth and online interventions, because these differences can contribute to persistent disparities in health across social groups [104]. Second, we were only able to measure until 10 weeks after birth. While we captured the first stressful weeks after birth including the infant crying peak, it could be that potential effects on parental distress levels or our measures of caregiving quality, including breastfeeding and room-sharing, become visible later on during the first year. Third, we did not measure parental use of external support services, including the use and quality of external breastfeeding courses and support services. It might be that, as a result of the intervention, parents used these support services more often, or that differences in our outcome variables only become visible among groups of parents who have limited access to (high-quality) support services. Fourth, as stated earlier, it could also be that the intervention did not impact on parental distress, but on other measures we could not take into account in this study. Observations and physiological measurements of both parental and infant behavior could provide more fine-grained information about the potential effect of the intervention.

## Conclusion

The current study offered no evidence that a universal prevention program implemented during pregnancy, and aimed at both mothers and fathers, is effective in preventing symptoms of parenting stress, depression, or anxiety during the first 10 weeks after birth. Also, we found no evidence that the intervention enhanced the quality of parental caregiving. However, parents reported the intervention, especially the information booklet, to be of added value. Future research should detect whether this type of brief psychoeducation might be effective on other measures, samples or periods in time. While absence of evidence is not evidence of absence, it is also possible that a brief psychoeducational intervention aimed at providing information is not sufficient to improve parental well-being and caregiving quality, and other, more intensive, types of interventions are needed. Since parental distress symptomatology and parental caregiving quality after birth can affect infant development [16, 21, 65], detecting effective ways of intervening in an early stage -thus already during pregnancy- is of vital importance for both parent’s as well as children’s health and development.

## Data Availability

The datasets used and/or analysed during the current study are available from the corresponding author on reasonable request.

## Abbreviations

AAP: American Academy of Pediatrics
ECR: Experiences in Close Relationships Questionnaire
EPDS: Edinburgh Postnatal Depression Scale
HADS: Hospital Anxiety and Depression Scale
IMS: The Investment Model Scale
MLM: Multilevel Lineair Modeling
MPAS: Maternal Postnatal Attachment Scale
PSI: Parenting Stress Index
PSQI: Pittsburgh Sleep Quality Index
SES: Socioeconomic status
SIDS: Sudden Infant Death Syndrome
T: Time
WHO: World Health Organization

## Footnotes

1 At t2, one questionnaire measuring delivery characteristics was sent to both parents. One of the parents could fill in this questionnaire. For all participating fathers, a questionnaire was returned at t2 (but not necessarily filled in by the father).
